# Cotton Felt

**Published:** 1875-08

**Authors:** 


### COTTON FELT.

On page 461 of the twenty-first volume of HALL'S JOURNAL OF HEALTH, being the number for December, 1874, we gave our views upon an important topic connected with human health and comfort, in an article entitled "A Wholesome Bed." We wrote the article in accordance with a system which we adopted long ago, and for which we have been thanked thousands of times. That system is, simply, to look up the really meritorious things in New York and elsewhere, and, after educating ourselves to a knowledge of their merits, to describe them in our pages for the benefit of our thousands of readers. The world has good things and bad things, in great abundance; and we have long considered it our duty to winnow the chaff from the pure grain for the benefit of those who look with respect upon our opinions and act in accordance with our expressed judgment.

Our friends who perused the editorial alluded to will remember that we spoke in terms of decided commendation of the Cotton Felt Mattrasses and cushions made by Mr. H. D. Ostermoor, of No. 75 Pearl street, New York. Our judgment was that they were cleaner, sweeter, wholesomer and purer than curled hair goods, while being a good deal cheaper and fully as durable. This was the testimony of trustworthy men and women of our acquaintance, confirmed by a very critical examination on our own part, of mattrasses and cushions long in wear.

A friend expressed to us a doubt as to the possibility of so preparing cotton as to prevent matting or hardening into lumps, in a cushion or mattrass after continued use. He would admit that nothing could be nicer than a cotton bed or cushion at first, but he felt certain that prolonged use would render it uneven and uncomfortable. So we sought additional information, and obtained evidence of the most satisfactory character. We explained to Mr. Ostermoor the doubts which our friend had expressed, and he cheerfully seconded our efforts towards more light. He has handed us the following letters: the first from Rev. Dr. Bellows, and the second from Rev. Mr. McArthur, both of which we gladly print.

NEW YORK, 232 E. 15th St.
June 7, 1875.

MR. H. D. OSTERMOOR—*Dear Sir:* I am happy to say that the Patent Felt Cushions, placed in our church by your company *twenty years ago* are in *excellent condition at this date.* Still *Elastic*, but little injured by moth, and *apparently likely to outlast most of those who sit upon them.*

We placed in the gallery of our church, *a dozen* years ago, when it was first erected, a certain number of *hair cushions*, meant to be of *first quality*, which are *not in as good condition as* their elders, the felt cushions in the body of the church. If your company continues to make the same article you did, I know of no reason why it ought not to supersede hair or any other known substance. I have recommended the felt cushions to many church building committees, as cheaper, better, free from danger from moth or tendency to mat and lose elasticity. Respectfully yours,

(Signed), HENRY W. BELLOWS,
*Pastor of all Soul's Church.*

NEW YORK, June 15th, 1875.

H. D. OSTERMOOR—*Dear Sir:* It gives me pleasure to say that the Patent Elastic Felt Cushions put by your company into the "*Calvary Baptist Church," more than twenty-one years ago, are still in excellent condition.* They still retain their elasticity—are free from moth or vermin of every sort. They have preserved their shape better, and are in every way more desirable than cushions made of any other material offered to the public. These cushions were put into the church long before I became a preacher. Of their present condition I can judge. In regard to the length of time they have been in use, and the satisfaction which they have uniformly given the officers of the church are my authority.

Truly yours,

(Signed), R. S. MCARTHUR,
*Pastor Calvary Baptist Church.*
*New York City.*

---

It would seem as if no further evidence need be adduced, so far as the freedom from "matting" tendencies is concerned. That ordinary cotton will become lumpy and matted after a while, we know; that this "Cotton Felt" will not, we are convinced. If this material in a cushion will stand the wear and tear of a score of years uninjured, it is probable that it would not be very seriously damaged by long use as a bed. But as beds are more frequently in use than church cushions, and as they are subject to somewhat different influences, we considered it wise to secure some additional testimony in that direction.

The evidence of a well known hotel proprietor, Mr. Howland, of Long Branch, was that he had used these mattrasses for more than twenty-one years, to his entire satisfaction. Mr. Morris of the same place, who is quite as widely known as a hotel man, used these words: "I have now in use Patent Elastic Felt mattrasses, which I bought in 1854, and they still retain their elasticity remarkably well. I consider them superior to hair or any other material in use. My hair mattrasses that I purchased at the same time, I have had re-made twice, and they at the present time are not as elastic as the Felt."

All this trustworthy testimony, added to the reasonable judgment which we ourselves are able to pass upon the subject, impels us to fully endorse this delightful substance, which is, it would appear, better in all respects than hair, while being also cheaper and more lasting.

---

TO REMOVE GREASE-SPOTS.—Thoroughly saturate the spot with benzine; then place a thick piece of blotting paper underneath the stain and another piece above it. A hot flat-iron is now applied to the upper piece of paper, and pressed on it for some time. The result is the complete absorption of the grease by the blotting-paper.

---

TO CLEAN GOLD CHAINS.—Put the chain in a small glass bottle with warm water, a little pulverized chalk, and some castile soap. Cork the bottle and shake it for a minute violently. The friction against the glass polishes the gold, and the soap and chalk extract every particle of grease and dirt from the interstices of a chain of the most intricate pattern. Rinse it in clear cold water, wipe with a towel, and the polish will surprise you.

